# Whole-Genome Sequencing Reveals the Resistome, Virulome, Pan-Genome Architecture, and Phylogenomic Diversity of Extensively Drug-Resistant *Salmonella enterica* Serovar Typhi in Pakistan

**DOI:** 10.3390/microorganisms14102225

**Published:** 2026-10-02

**Authors:** Rabia Mehr, Qaisar Ali, Muhammad Zahid, Taj Ali Khan, Muhammad Asghar, Noor Rehman, Taane G. Clark, Reaz Uddin, Ajmal Khan

**Affiliations:** 1 Department of Zoology, Islamia College Peshawar, Peshawar 25120, Pakistan; 2 Center of biotechnology and microbiology, University of Peshawar, Peshawar 25120, Pakistan; 3 Institute of Pathology and Diagnostic Medicine, Khyber Medical University, Peshawar 25100, Pakistan; 4 Public Health Reference Laboratory (PHRL), Khyber Medical University (KMU), Phase 5, Hayatabad, Peshawar 25000, Pakistan; 5 Department of Pathology, Khyber Teaching Hospital, Khyber Medical College, Peshawar 25120, Pakistan; 6 Faculty of Infectious and Tropical Diseases, London School of Hygiene and Tropical Medicine, London WC1E 7HT, UK; 7 Faculty of Epidemiology and Population Health, London School of Hygiene and Tropical Medicine, London WC1E 7HT, UK; 8 Dr. Panjwani Center for Molecular Medicine and Drug Research, International Center for Chemical and Biological Sciences, University of Karachi, Karachi 75270, Pakistan; 9 Natural and Medical Sciences Research Center, University of Nizwa, PO Box 33, Nizwa 616, Oman; 10 Department of Chemical and Biological Engineering, College of Engineering, Korea University, Seoul 02841, Republic of Korea

**Keywords:** *Salmonella enterica* serovar Typhi, antimicrobial resistance, multidrug resistance, extensively drug-resistant, whole-genome sequencing, resistome, virulome, phylogenomics

## Abstract

The emergence of multidrug-resistant (MDR) and extensively drug-resistant (XDR) *Salmonella enterica* serovar Typhi has severely constrained typhoid treatment. Pakistan is a major hotspot for XDR typhoid, yet genomic evidence defining the molecular basis of antimicrobial resistance, virulence, and genomic diversity of circulating strains remains limited. A cross-sectional study was conducted from January to December 2021 involving 252 culture-confirmed *S. Typhi* isolates collected from patients with suspected typhoid fever in Peshawar, Pakistan. Isolates were identified by conventional microbiological methods, biochemical testing, and 16S rRNA PCR. Antimicrobial susceptibility testing (AST) was performed according to CLSI guidelines, and isolates were classified as susceptible, MDR, or XDR following WHO criteria. Fourteen non-duplicate XDR isolates were selected for Illumina whole-genome sequencing (WGS). Bioinformatic analyses included genome assembly, multilocus sequence typing (MLST), resistome and virulome characterization, mutation analysis, pan-genome analysis, and core-genome phylogenetic reconstruction. Pearson’s chi-square goodness-of-fit test was used to assess categorical distributions, with *p* < 0.05 considered statistically significant. Among the 252 isolates, 152 (60.3%) were XDR, 73 (29.0%) were MDR, and 27 (10.7%) were susceptible. The distributions of sex, age group, and phenotypic classification differed significantly from equal expected proportions (*p* = 0.001, *p* = 0.013, and *p* < 0.001, respectively). Resistance was highest to ciprofloxacin, ampicillin, chloramphenicol, ceftriaxone, and co-Trimoxazole, whereas azithromycin and meropenem showed high in vitro activity. WGS showed that all sequenced isolates belonged to sequence type ST1 and had conserved genome sizes (4.84–4.95 Mb) with ~54% GC content. The resistome included *blaCTX-M-15*, *blaTEM-1*, *catA1*, *sul1*, *qnrS1*, *aac(6′)-Iy*, and *dfrA7*, with mutations in *gyrA*, *parC*, *soxS*, *pmrA*, and *pmrB*. Virulence genes associated with adhesion, type III secretion, Vi capsule biosynthesis, intracellular survival, iron acquisition, and typhoid toxin were conserved across all isolates. Pan-genome analysis indicated limited pan-genome expansion within the sequenced collection, while phylogenomic analysis showed high genetic relatedness among the sequenced isolates, indicating substantial genomic conservation within this collection. The high genomic relatedness and limited gene-content variation observed within the 14 sequenced XDR isolates support a genetically conserved collection, but do not by themselves establish ongoing transmission or clonal expansion. Larger and more geographically diverse genomic datasets are needed to determine the broader population structure of XDR *S. Typhi* in Pakistan. Continued genomic surveillance, appropriate antibiotic use, typhoid conjugate vaccination, and effective infection-control measures are needed to reduce transmission of drug-resistant typhoid.

## 1. Introduction

*Salmonella enterica* serovar Typhi, the causative agent of typhoid fever, is a major cause of bloodstream infection in low- and middle-income countries, with an estimated 11–21 million infections and 128,000–161,000 deaths annually, particularly in South Asia and sub-Saharan Africa [1]. Transmission occurs primarily through ingestion of food or water contaminated with human fecal material and is associated with inadequate sanitation, unsafe drinking water, and poor food hygiene [2,3]. Pakistan continues to experience a substantial burden of typhoid fever, compounded by the emergence and dissemination of antimicrobial-resistant *S. Typhi*. Ampicillin, chloramphenicol, and trimethoprim-sulfamethoxazole were previously used as first-line therapies; however, the emergence of multidrug-resistant (MDR) *S. Typhi* during the 1980s and 1990s prompted increased reliance on fluoroquinolones and third-generation cephalosporins [4,5]. Subsequent resistance to these agents led to the emergence of extensively drug-resistant (XDR) *S. Typhi*, defined by resistance to first-line antibiotics, fluoroquinolones, and third-generation cephalosporins [6]. The first major outbreak of XDR *S. Typhi* was reported in Sindh, Pakistan, in 2016, followed by its spread to other regions of the country [7], further narrowing therapeutic options and increasing concerns regarding treatment failure and international dissemination [8].

The evolution of AMR in *S. Typhi* results from chromosomal mutations and acquisition of resistance genes carried by mobile genetic elements such as plasmids, transposons, and integrons [9]. Fluoroquinolone resistance is associated with mutations in the quinolone resistance-determining regions of *gyrA* and *parC*, with additional changes reported in *gyrB* and *parE* [10]. Resistance to third-generation cephalosporins is mediated by ESBL genes, particularly *blaCTX-M-15*, while other determinants, including *blaTEM-1*, *catA1, sul1, dfrA7*, and *qnrS1*, contribute to resistance to *β*-lactams, chloramphenicol, sulfonamides, trimethoprim, and fluoroquinolones, respectively [11]. Alterations in regulatory pathways and increased efflux activity may further contribute to reduced antimicrobial susceptibility [12]. Beyond AMR, *S. Typhi* carries multiple virulence determinants that support intestinal colonization, epithelial invasion, intracellular survival, immune evasion, and systemic infection [13]. These include *Salmonella* pathogenicity islands (SPIs) encoding Type III secretion systems (T3SS), fimbrial and non-fimbrial adhesins involved in host–cell attachment, the Vi capsular polysaccharide involved in immune evasion, iron acquisition systems, magnesium transport proteins, and genes associated with intracellular survival [14]. The typhoid toxin, encoded by *cdtB, pltA*, and *pltB*, contributes to disease pathogenesis and systemic effects [15]. The coexistence of AMR and virulence determinants in the same strains may facilitate persistence and transmission, emphasizing the need for genomic analysis of circulating *S. Typhi* populations [16].

Whole-genome sequencing (WGS) has improved the surveillance of bacterial pathogens by allowing simultaneous analysis of genomic variations, sequence types (STs), antimicrobial resistance, virulence determinants, and genetic relatedness [17]. Unlike conventional typing methods, WGS can resolve strain-level differences and provide information on transmission, population structure, and resistance mechanisms [18]. Approaches including multilocus sequence typing (MLST), resistome and virulome analyses, pan-genome analysis, and phylogenomic reconstruction are therefore useful for characterizing drug-resistant *S. Typhi* populations [19]. WGS provides enhanced resolution of the genetic diversity, population structure, and evolutionary relationships of circulating *S. Typhi* lineages beyond conventional MLST. Several studies have described the genomic features of XDR *S. Typhi* from Pakistan; however, important differences in sampling, geographic coverage, and analytical scope remain among available studies. In particular, the genomic characteristics of independently collected, temporally defined clinical XDR isolates from Peshawar require further characterization using integrated phenotypic and genome-wide analyses.

Previous genomic and epidemiological studies have provided important insights into the emergence and dissemination of antimicrobial-resistant *S. Typhi* in Pakistan. Following the emergence of XDR *S. Typhi* in Sindh in 2016, subsequent studies have documented the presence of sequence type ST1 and associated resistance determinants in different regions of the country. Mumtaz et al. (2024) conducted WGS of 13 XDR *S. Typhi* isolates from the Peshawar region and reported a highly conserved genotypic background, with all isolates belonging to ST1 and carrying major resistance determinants, including *blaCTX-M-15*, *blaTEM-1*, *catA1*, *sul1*, *dfrA7*, and *qnrS1* [20]. Their broader pan-genome analysis, incorporating 140 XDR genomes from Pakistan, further demonstrated limited genomic diversity among these isolates. Similarly, Dar et al. (2026) analyzed 71 publicly available *S. Typhi* genomes from Pakistan and identified ST1 and ST2 as the predominant sequence types, with plasmid replicons detected in a proportion of isolates [21]. Epidemiological surveillance in Khyber Pakhtunkhwa has also demonstrated a substantial burden of antimicrobial resistance (AMR); Ahmad et al. (2025) reported MDR and XDR phenotypes among *S. Typhi* isolates from the province, with Peshawar showing the highest prevalence [22]. In addition, Fatima et al. (2023) identified ST1-associated XDR strains carrying the IncQ1 plasmid in Balochistan, supporting the wider occurrence of multidrug-resistant *S. Typhi* lineages in Pakistan [23].

Although these studies have established the widespread occurrence of ST1-associated XDR *S. Typhi* and major resistance determinants in Pakistan, the present study addresses a more specific gap by analyzing an independently collected, temporally defined 2021 clinical isolate collection from Peshawar and integrating phenotypic AST with genome-wide characterization of resistance determinants, virulence-associated genes, plasmid content, pan-genome structure, and core-genome phylogenetic relationships. Importantly, rather than using ST1 assignment as evidence of clonality, we evaluate genomic relatedness using core-genome phylogeny and single nucleotide polymorphism (SNP)-level variations. This integrated analysis provides an independent assessment of the genomic conservation and diversity of XDR *S. Typhi* circulating in Peshawar and allows comparison with previously characterized Pakistani isolates.

Therefore, the present study combined antimicrobial susceptibility testing (AST) with WGS to characterize AMR *S. Typhi* isolates from patients with typhoid fever in Peshawar, Pakistan. We determined the prevalence of MDR and XDR isolates, identified resistance and virulence determinants, assessed sequence types and core-genome phylogenetic relationships and SNP-level variations, and characterized pan-genome diversity among XDR isolates.

## 2. Materials and Methods

### 2.1. Study Design, Sample Collection, and Bacterial Isolation

A cross-sectional study was conducted from January to December 2021 at the Department of Zoology, Islamia College, Peshawar and Centre of Biotechnology and Microbiology, University of Peshawar, Pakistan, in collaboration with the Department of Pathology, Khyber Teaching Hospital (KTH), Peshawar. During the study period, blood specimens were collected from 252 patients presenting with clinical features that were suggestive of enteric fever. Blood cultures were performed using the Thermo Scientific^TM^ SIGNAL Blood Culture System (Thermo Fisher Scientific, Basingstoke, UK) according to the manufacturer’s protocols and yielded positive results. Culture-positive specimens were subcultured onto blood agar, MacConkey agar, and Salmonella–Shigella agar followed by aerobic incubation at 37 °C for 24 h. Presumptive Salmonella isolates were identified based on colony morphology, Gram-staining characteristics, and biochemical profiling using the API 20E identification system (bioMérieux, Marcy-l’Étoile, France) [24,25,26].

### 2.2. Genomic DNA Extraction and 16S rRNA Gene Amplification

Genomic DNA was extracted from overnight bacterial cultures using the GeneJET Genomic DNA Purification Kit (Thermo Fisher Scientific, Waltham, MA, USA) following the manufacturer’s protocol. DNA concentration and purity were quantified using the Qubit^®^ 2.0 Fluorometer (Thermo Fisher Scientific, Waltham, MA, USA). Presumptive *Salmonella* isolates were subjected to PCR amplification of the bacterial 16S rRNA gene to assess bacterial DNA quality and support molecular identification before downstream analyses (primer details are in Supplementary Table S1). PCR products were run by agarose gel electrophoresis and visualized under ultraviolet illumination to verify amplification of the expected amplicon [27,28]. Thus, 252 culture-positive *Salmonella* isolates were included in the phenotypic antimicrobial susceptibility analysis, while 14 non-duplicate XDR *S. Typhi* isolates were subsequently selected for whole-genome sequencing.

### 2.3. Antimicrobial Susceptibility Testing (AST)

AST was performed using the Kirby–Bauer disk diffusion method on Mueller–Hinton agar as per the *Clinical and Laboratory Standards Institute (CLSI) M100, 30th edition (2020)* guidelines [7]. Fresh bacterial cultures were adjusted to 0.5 McFarland of turbidity before inoculation. The antibiotic disks used included ampicillin (10 µg), chloramphenicol (30 µg), co-trimoxazole (1.25/23.75 μg), ciprofloxacin (5 μg), ceftriaxone (30 μg), azithromycin (15 μg), and meropenem (10 μg) (Oxoid Ltd., Basingstoke, UK). Inhibition zone diameters were interpreted according to the applicable CLSI breakpoints, and results were recorded separately as susceptible (S), intermediate (I), or resistant (R). A sensitivity analysis was performed to assess the effect of alternative handling of intermediate results on antimicrobial resistance frequencies and MDR/XDR classification. Quality control was performed using *Salmonella enterica* serovar Typhimurium ATCC 14028 as the reference strain [29]. MDR isolates were defined according to the WHO recommendations as isolates resistant to the three traditional first-line antimicrobial agents (ampicillin, chloramphenicol, and trimethoprim–sulfamethoxazole). XDR *S. Typhi* was defined as isolates that were resistant to ampicillin, chloramphenicol, trimethoprim-sulfamethoxazole, fluoroquinolones, and third-generation cephalosporins [23].

### 2.4. Selection of Isolates for Whole-Genome Sequencing

Fourteen non-duplicate XDR *S. Typhi* isolates were selected for WGS to represent the XDR isolates recovered during the study period. Selection was based on the availability of high-quality genomic DNA and distinct patient samples.

### 2.5. Whole-Genome Sequencing

High-quality genomic DNA was subjected to paired-end sequencing using the Illumina HiSeq platform (Illumina, Inc., San Diego, CA, USA). Raw sequencing reads were quality-assessed before downstream analyses, and low-quality bases and adapter sequences were removed using FastQC v0.12.1 and fastp v0.23.4, respectively [30]. Sequencing quality and genome coverage metrics, including total reads, mean sequencing depth, percentage genome coverage, and quality-filtered reads, were recorded for each isolate.

### 2.6. Genome Assembly, Annotation, and Quality Assessment

High-quality reads were assembled de novo using SPAdes v3.15.5 (Center for Algorithmic Biotechnology, St. Petersburg, Russia), and assembly quality was evaluated using QUAST v5.2.0 (Center for Algorithmic Biotechnology, St. Petersburg, Russia). Draft genome annotation was performed using Prokka v1.14.6 and further validated using the Rapid Annotation using Subsystem Technology (RAST) server. Assembly quality was assessed using the genome size, GC content, N50 values, and contig statistics before inclusion in comparative genomic analyses [30].

### 2.7. Sequence Typing and Comparative Genomic Analyses

Multilocus sequence typing (MLST) was performed using MLST v2.23.0 (https://github.com/tseemann/mlst (accessed on 11 November 2025)) to assign a sequence type (ST) to each sequenced *S. Typhi* isolate. The analysis was based on the allelic profiles of the seven housekeeping genes defined in the *Salmonella* MLST scheme, allowing the isolates to be assigned to established sequence types and compared according to their clonal backgrounds [31]. Antimicrobial resistance (AMR) determinants were characterized from the assembled genomes using four complementary approaches. ResFinder v4.6.0 (https://cge.food.dtu.dk/services/ResFinder/ (accessed on 20 November 2025)) was used to identify acquired antimicrobial resistance genes [32]. The Comprehensive Antibiotic Resistance Database (CARD) v3.2.9 (https://card.mcmaster.ca/ (accessed on 20 November 2025)) and its Resistance Gene Identifier (RGI) were used to identify resistance genes and associated resistance mechanisms [33]. AMRFinderPlus v3.12 (https://github.com/ncbi/amr (29 November 2025) was additionally used to detect AMR genes and relevant resistance-associated determinants [33]. Chromosomal resistance-associated point mutations were identified using PointFinder v4.1 (Center for Genomic Epidemiology, Technical University of Denmark, Lyngby, Denmark) [34]. Results from these tools were compared to obtain a comprehensive profile of acquired AMR genes and chromosomal mutations in each isolate. Virulence-associated genes were screened using ABRicate v1.0.1 (https://github.com/tseemann/abricate (accessed on 3 December 2025)) against the Virulence Factor Database (VFDB) (http://www.mgc.ac.cn/VFs/ (accessed on 3 December 2025)) [35,36]. The analysis was used to determine the presence and distribution of genes associated with *S. Typhi* virulence and pathogenicity among the sequenced isolates. Plasmid-associated replicons were identified using PlasmidFinder v2.1 (https://cge.food.dtu.dk/services/PlasmidFinder/ (accessed on 3 December 2025)) to assess the presence of known plasmid replicon types in the assembled genomes [36]. The resulting MLST, AMR, virulence, and plasmid profiles were compiled and compared across isolates to evaluate genomic similarities and differences within the sequenced XDR *S. Typhi* collection.

### 2.8. Pan-Genome and Phylogenomic Analyses

Pan-genome analysis was conducted using Roary v3.13.0 (-e -n) to generate a MAFFT-based core-gene alignment from GFF3 annotations of all clinical isolates and four international reference genomes. Core-genome phylogenetic relationships were reconstructed using IQ-TREE v3.0.1 under maximum-likelihood inference with 1000 ultrafast bootstrap replicates. ModelFinder selected HKY+F with uniform rate heterogeneity as the best-fit substitution model according to BIC. The tree was rooted using *S. Typhi* CT18 as the outgroup, reflecting its status as the earliest characterized and most divergent reference genome in the alignment. Four international reference genomes were included for context: CT18 (GenBank AL513382), a foundational, globally referenced *S. Typhi* genome; 154NSRL (RefSeq NZ_CP169078.1), a 2022 cephalosporin-resistant clinical isolate from Ahmedabad, India; and two travel-associated genomes linked to Pakistan: R19.2839 (RefSeq NZ_CP046429.1), a multidrug-resistant isolate characterized by the Taiwan Centres for Disease Control, and WGS1146 (RefSeq NZ_CP040575.1), a ceftriaxone-resistant isolate recovered from a pregnant traveler returning from Pakistan to Denmark in 2019. These genomes were selected to place the clinical isolates in this study within the context of recently characterized MDR/XDR *S. Typhi* lineages with documented links to South Asia. Trees were visualized and annotated using the Interactive Tree of Life (iTOL v6) [28]. As a sensitivity analysis, recombinant regions in the core-genome alignment were identified using Gubbins v2.3.4. Phylogenetic reconstruction was subsequently performed using a recombination-masked alignment to assess the robustness of phylogenetic relationships to recombination. Given the limited number of informative sites remaining after masking, the full core-genome alignment was retained for the primary phylogenetic analysis [20].

### 2.9. Statistical Analysis

Statistical analyses were performed using IBM SPSS Statistics v23.0 (IBM Corp., Armonk, NY, USA). Demographic characteristics and AMR profiles were summarized using descriptive statistics. Pearson’s chi-square goodness-of-fit test was used to determine whether the observed distributions of categorical variables differed from equal expected proportions. A two-tailed *p*-value < 0.05 was considered statistically significant. No adjustment for multiple testing was applied because these analyses were descriptive and exploratory.

## 3. Results

### 3.1. Clinical Characteristics and Distribution of S. Typhi Isolates

A total of 252 *S. Typhi* isolates were recovered from patients with suspected typhoid fever (Table 1). The distribution by gender differed significantly from an equal distribution (χ^2^ goodness-of-fit, *p* = 0.001), with 150 (59.5%) isolates obtained from male and 102 (40.5%) from female patients. Patients aged 6–15 years accounted for the largest proportion of isolates (128/252, 50.8%), followed by those aged ≥16 years (72/252, 28.6%) and ≤5 years (52/252, 20.6%); the age group distribution also differed significantly from equal proportions (χ^2^ goodness-of-fit, *p* = 0.013). Phenotypic AST classified 152 (60.3%) isolates as XDR, 73 (29.0%) as MDR, and 27 (10.7%) as susceptible, with a significant difference in the distribution of phenotypic categories (χ^2^ goodness-of-fit, *p* < 0.001), (Table 1).

### 3.2. Antimicrobial Susceptibility Testing

**Figure 1:**
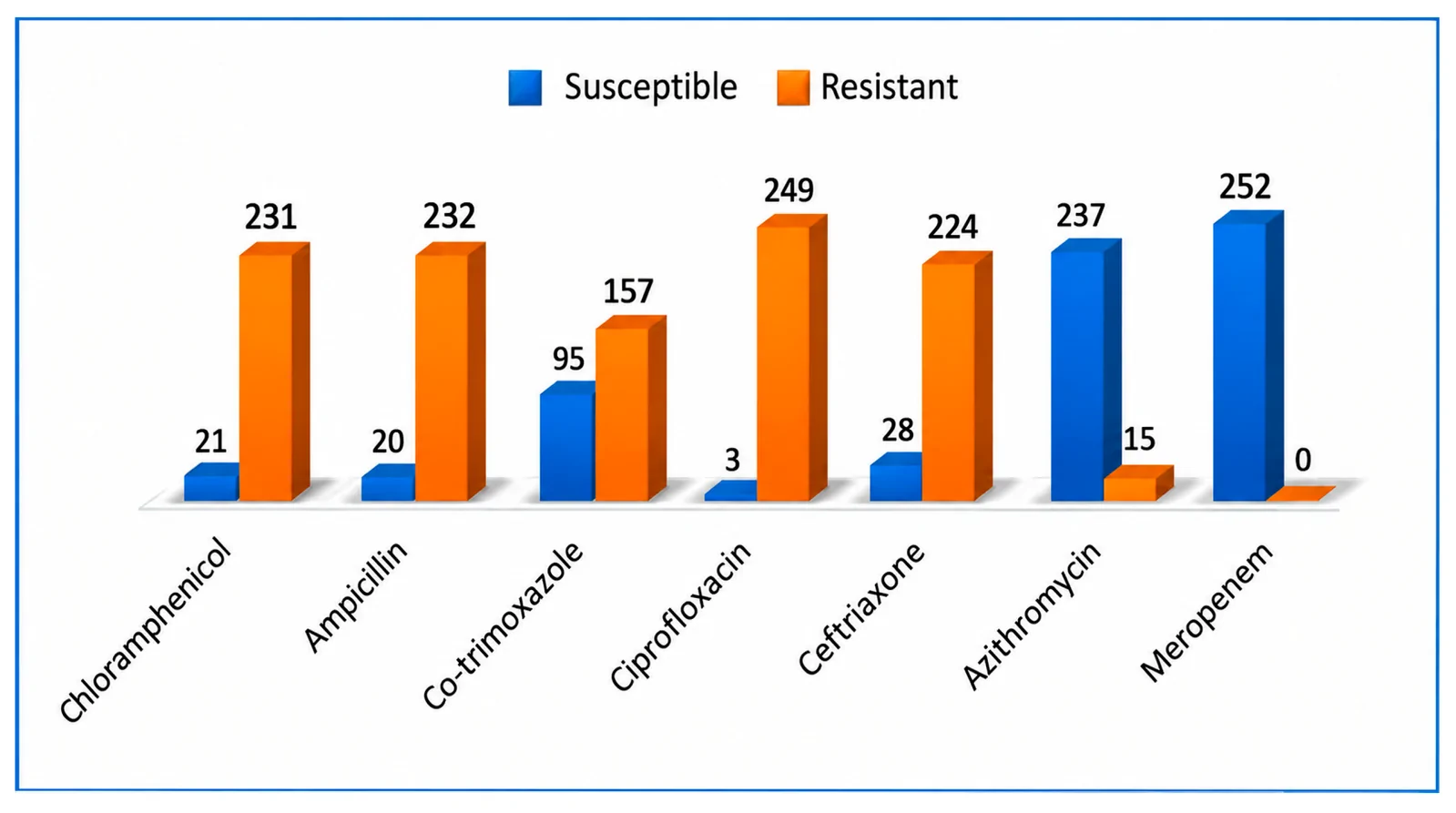
Antimicrobial susceptibility profile of 252 *Salmonella enterica* serovar Typhi isolates.

The phenotypic AST demonstrated a high prevalence of resistance to first- and second-line antimicrobial drugs among the 252 *S. Typhi* isolates. Resistance was most frequently observed against ciprofloxacin (249/252, 98.8%), followed by ampicillin (232/252, 92.1%), chloramphenicol (231/252, 91.7%), ceftriaxone (224/252, 88.9%), and co-trimoxazole (157/252, 62.3%). In contrast, azithromycin and meropenem remained highly effective, with susceptibility rates of 94.0% (237/252) and 100% (252/252), respectively (Figure 1). The predominance of resistance to ampicillin, chloramphenicol, co-trimoxazole, ciprofloxacin, and ceftriaxone was consistent with the high proportion of isolates classified as XDR.

### 3.3. Genomic Characterization, Sequence Typing, and Resistome Profiling

**Figure 2:**
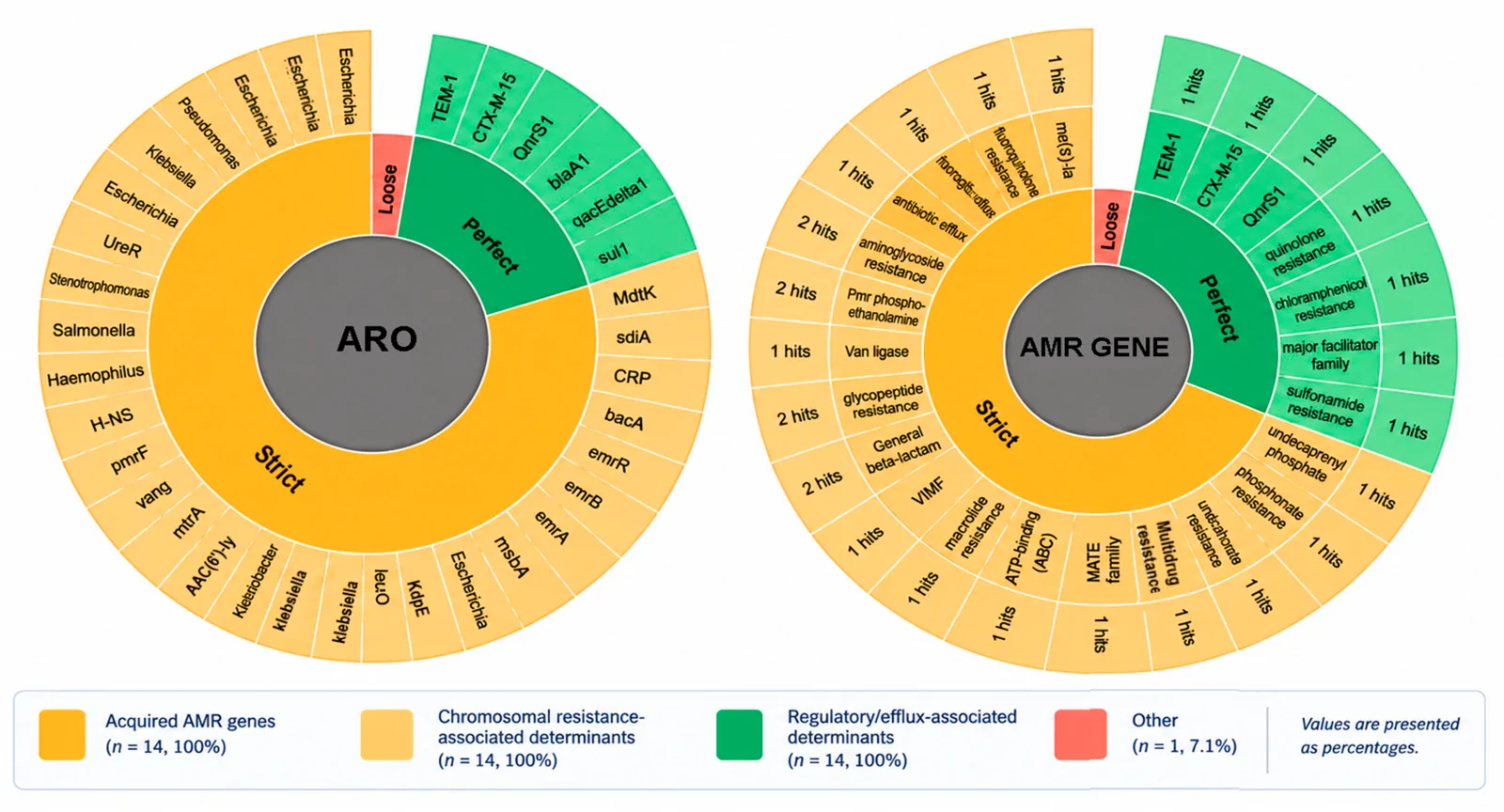
Antimicrobial resistance determinants identified using the Comprehensive Antibiotic Resistance Database (CARD) among 14 whole-genome sequenced XDR *Salmonella Typhi* isolates. The sunburst plots show CARD annotations according to resistance determinants and associated mechanisms. Inner rings indicate Perfect, Strict, and Loose matches, while outer segments represent individual resistance determinants; “hits” indicate the number of detected matches.

WGS was performed on 14 non-duplicate XDR *S. Typhi* isolates selected on the basis of their identical phenotypic resistance profiles. MLST analysis assigned all 14 sequenced isolates to sequence type ST1, indicating the circulation of a genetically homogeneous lineage within the study population. Plasmid analysis did not identify any detectable plasmid replicons in the sequenced genomes. Comprehensive resistome analysis identified a highly conserved repertoire of AMR determinants across all sequenced isolates (Figure 2). Genes encoding resistance to *β*-lactams, aminoglycosides, fluoroquinolones, chloramphenicol, sulfonamides, and multidrug efflux systems were consistently detected. All genomes carried the extended-spectrum *β*-lactamase gene *blaCTX-M-15*, together with *bla*TEM-1, *catA1*, *acc(6′)-Iy*, *dfrA7*, *gyrA*, *gyrB*, *qnrS1*, *parC*, *parE*, *folP*, *SoxS*, *acrA*, *mdfA*, *emrB*, *emrR*, *AcrAB–TolC*, *pmrA*, and *pmrB*. These resistance determinants corresponded closely with the observed phenotypic resistance to ampicillin, chloramphenicol, co-trimoxazole, ciprofloxacin, and ceftriaxone. A small number of accessory resistance determinants exhibited limited variability among the sequenced isolates. The aminoglycoside resistance gene *aph(6)-Id* was identified in isolates ST36, ST42, and ST84, whereas the sulfonamide resistance gene *sul1* was detected only in isolates ST40 and ST84. Apart from these minor differences, the resistome composition remained highly conserved across all 14 genomes (Table 2).

### 3.4. Mutation Analysis of Antimicrobial Resistance-Associated Genes

Comparative genomic analysis identified several non-synonymous mutations in antimicrobial resistance-associated genes and regulatory determinants among the sequenced XDR *S. Typhi* isolates. A substitution in the global regulatory gene soxS resulted in an aspartic acid-to-asparagine change at amino acid position 19 (p.D19N). In the quinolone resistance-determining region, the *gyrA* gene contained an alanine-to-threonine substitution at position 119 (p.A119T), while the *parC* gene harbored a p.Phe173Ile substitution. The *pmrA* gene contained two amino acid substitutions, p.G211R and p.T89S, whereas the *pmrB* gene exhibited three substitutions, p.G73S, p.I83V, and p.M15T, which have previously been associated with reduced polymyxin susceptibility. The distribution of all identified amino acid substitutions is summarized (Table 3).

### 3.5. Virulence Gene Profiling

Virulence profiling demonstrated a highly conserved distribution of major virulence determinants among all XDR *S. Typhi*. Genes encoding fimbrial adhesion, including the *csg*, *bcf*, and *fim* gene clusters, were detected in every genome. Likewise, genes associated with both *Salmonella Pathogenicity Island 1 (SPI-1)* and *Salmonella Pathogenicity Island 2 (SPI-2)* type III secretion systems were consistently identified, including components of the *inv*, *org*, *prg*, *spa*, *ssa*, *ssc*, *sse*, *ssr*, *sip*, *pip*, and *sif* gene families.

All isolates also possessed the *vexABCDE* locus associated with Vi capsular polysaccharide expression, with the *vex* genes primarily representing components involved in Vi capsule export and transport. Additional conserved virulence determinants included the non-fimbrial adhesion gene *misL*, the macrophage survival gene *mig-14*, magnesium transport genes *mgtB* and *mgtC*, and the iron acquisition gene *iroN*. The typhoid toxin-associated gene *cdtB* was detected in all sequenced isolates; *cdtB* forms part of the typhoid toxin complex together with *pltA* and *pltB*. Overall, the virulence gene repertoire was highly conserved across all 14 genomes, with no substantial differences observed in the distribution of the major virulence-associated gene clusters (Table 4).

### 3.6. Functional Annotation and COG Distribution Analysis

**Figure 3:**
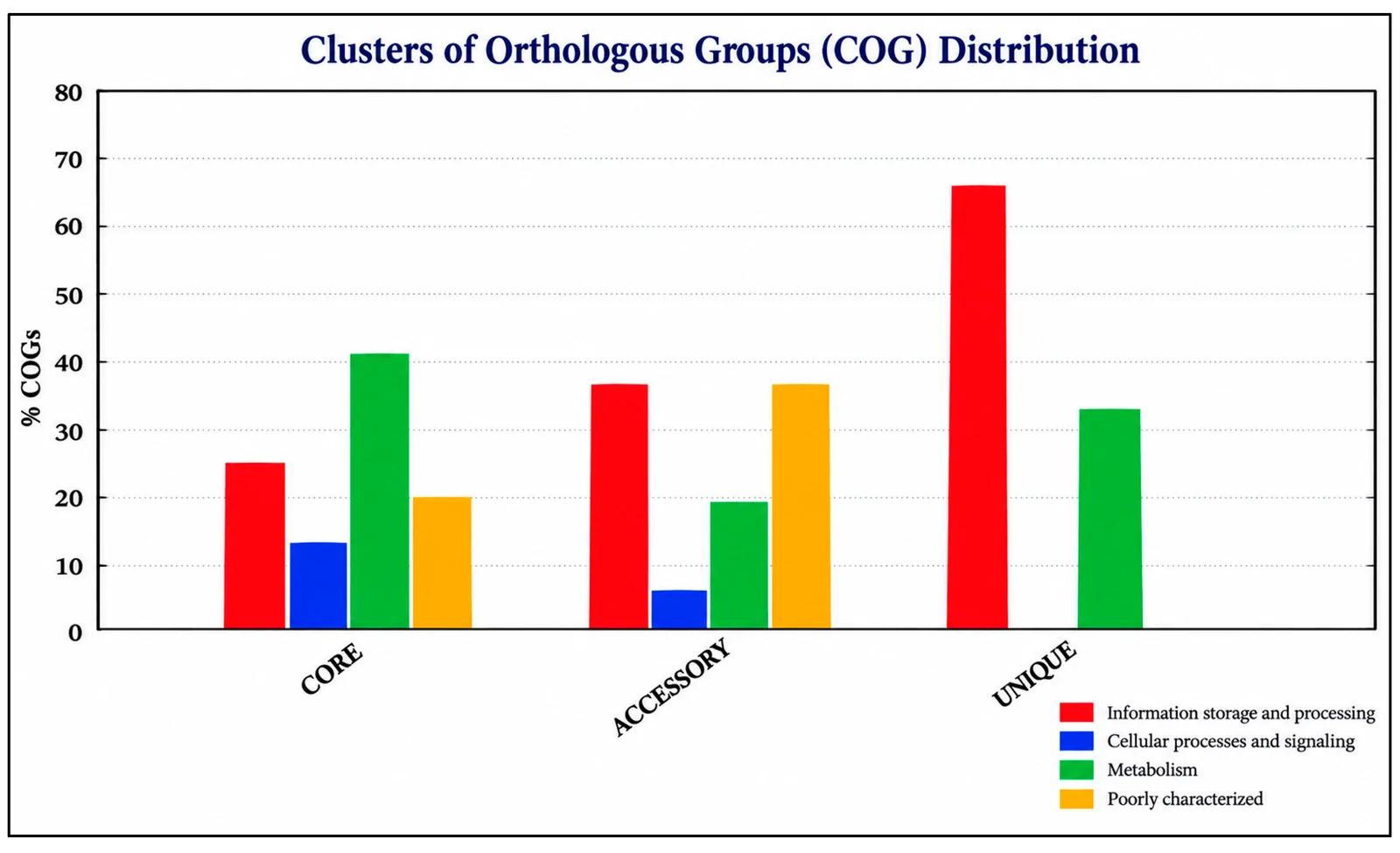
Functional distribution of core, accessory, and unique genes among XDR *S. Typhi* isolates based on Clusters of Orthologous Groups (COG) classification. Core genes were predominantly associated with metabolism (41%), whereas accessory genes were mainly assigned to information storage and processing and poorly characterized functions (37% each). Unique genes were primarily associated with information storage and processing (66%) and cellular processes and signaling (33%).

Clusters of Orthologous Groups (COG) analysis was performed to characterize the functional composition of the core, accessory, and unique genomes. Functional annotation based on KEGG database mapping demonstrated distinct distributions of COG categories among the three genomic compartments (Figure 3). Core genes accounted for 12–40% of the annotated genome and were assigned to essential cellular functions, including central metabolism, energy production, transcription, translation, and DNA replication. Accessory genes comprised 5–39% of the genome and were primarily associated with metabolic pathways and enzymatic activities. In contrast, more than 65% of the unique genes were classified within categories related to information storage and processing. Approximately one-third of genes (~32%) across all genomic compartments were assigned to poorly characterized functional categories, reflecting the presence of proteins with predicted or currently unknown biological functions (Figure 3).

### 3.7. Functional Characterization of the Pan-Genome

**Figure 4:**
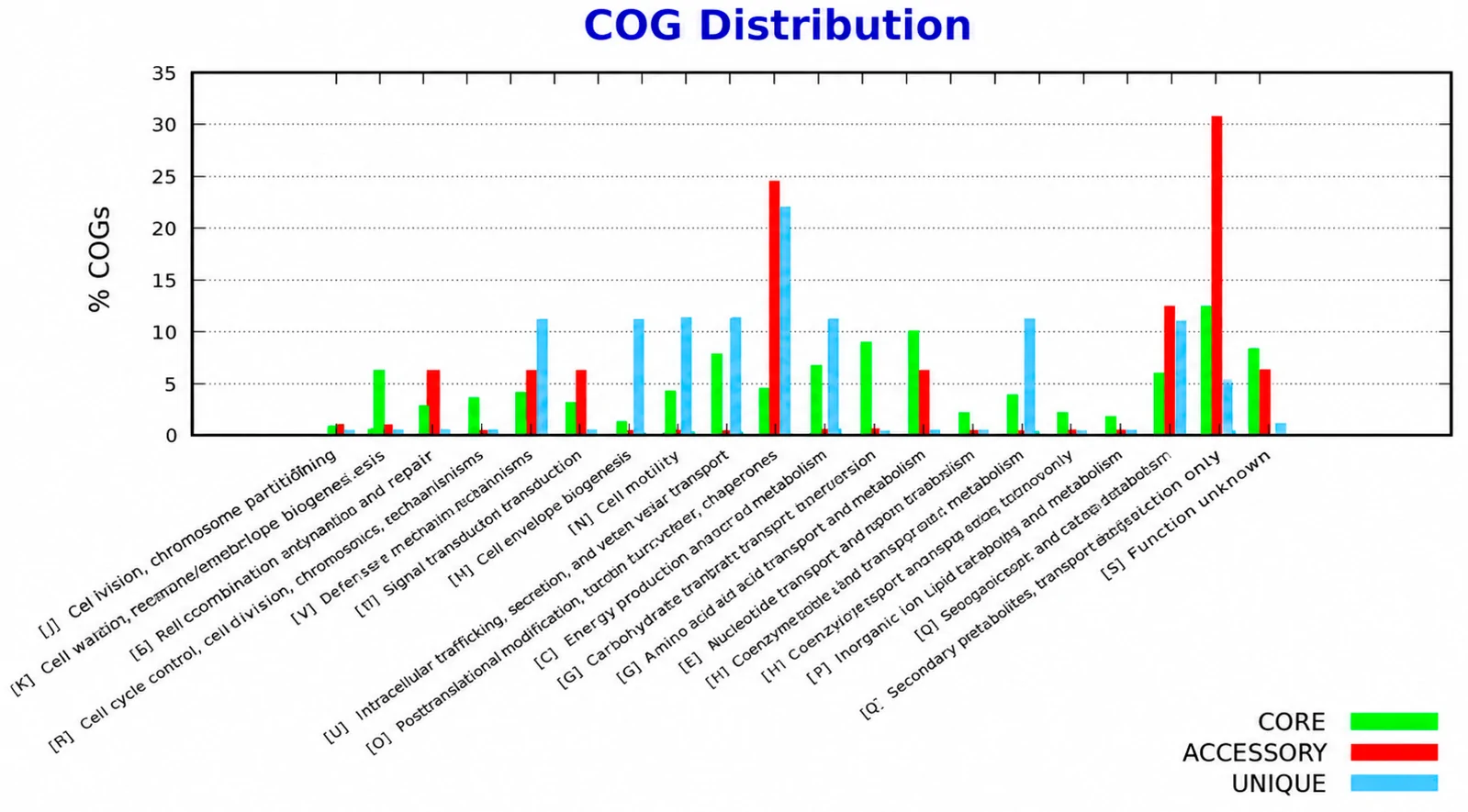
The functional distribution of genes within the pan-genome, categorized according to COG (Clusters of Orthologous Groups) classifications. The functional assignment of proteins encoded by core (green), auxiliary (red), and unique (blue) genes is shown in the histogram. This functional stratification highlights the evolutionary pressure to conserve core biological functions while allowing diversity in genes associated with niche adaptation and survival strategies.

Functional categorization of the pan-genome further demonstrated distinct patterns of gene distribution across the core, accessory, and unique genomic fractions (Figure 4). Core genes were enriched in conserved cellular processes, including translation and ribosomal biogenesis (J); transcription (K); DNA replication, recombination, and repair (L); and energy production and conversion (C). Accessory genes showed greater representation in functional categories associated with carbohydrate metabolism (G), amino acid transport and metabolism (E), and coenzyme metabolism (H). Unique genes were principally assigned to replication, recombination, and repair (L); transcription (K); and signal transduction mechanisms (T). A substantial proportion of unique genes also belonged to the poorly characterized functional categories R (general functional prediction only) and S (function unknown). The overall functional distribution highlighted clear differences between conserved housekeeping functions encoded by the core genome and the more variable functional repertoire represented within the accessory and unique genomes (Figure 4).

### 3.8. Core- and Pan-Genome Analyses

**Figure 5:**
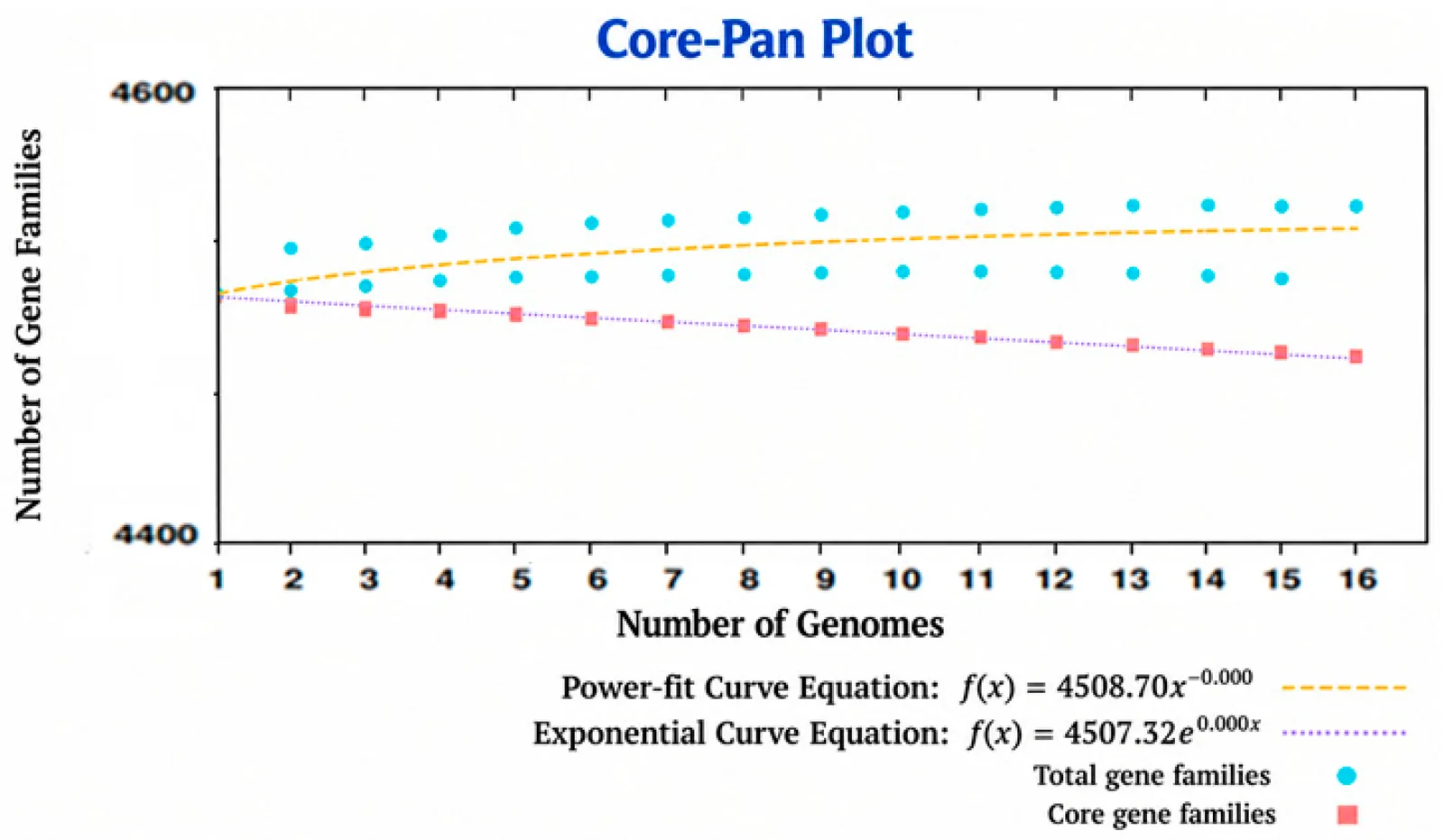
Core- and pan-genome analyses of the sequenced XDR *S. Typhi*. The accumulation curves illustrate changes in the number of core- and pan-genome gene families with sequential genome addition. Power-law and exponential model fitting indicate a limited pan-genome expansion within the sequenced collection characterized by extensive conservation of chromosomal gene content.

Core- and pan-genome analyses were performed to evaluate the genomic diversity of study isolates. Progressive inclusion of additional genomes resulted in a gradual stabilization of the core genome and only a minimal increase in the number of newly identified gene families, suggesting a limited pan-genome expansion within the sequenced isolates. Model fitting supported this genomic structure, with both the power-law model (f(*x*) = 4508.70*x*^−0.000^) and exponential model (f(*x*) = 4507.32^e0.000*x*^) demonstrating minimal expansion of the pan-genome as additional genomes were incorporated. These findings indicate a high degree of genomic conservation among the sequenced isolates (Figure 5).

### 3.9. Phylogenomic Analysis

**Figure 6:**
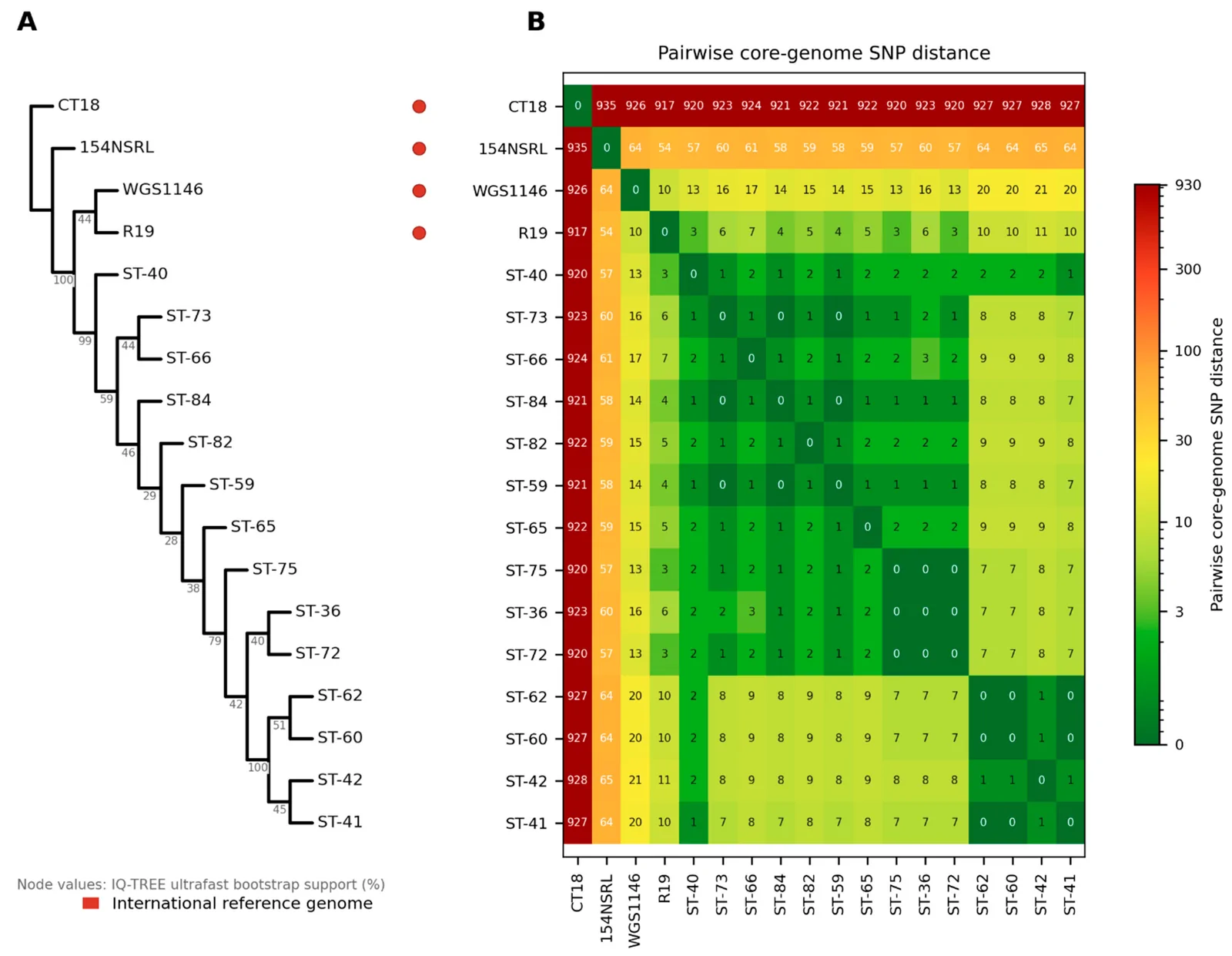
Phylogenomic relationships and pairwise genomic distances among *S. Typhi* isolates. (**A**) Cladogram derived from the core-genome maximum-likelihood phylogeny; topology reflects the inferred evolutionary relationships, but branch lengths are not drawn to scale. Node values indicate IQ-TREE ultrafast bootstrap support (%). International reference genomes are indicated by a red dot. (**B**) Heatmap of pairwise core-genome SNP distances between all isolates and reference genomes, in the same row order as panel (**A**); cell values provide the exact SNP count for each pair. The color scale is logarithmic to accommodate the wide range of distances observed (0–935 SNPs). The red circle and rectangles show international reference genome. The full pairwise matrix is provided in Supplementary Table S7.

Maximum-likelihood phylogenetic analysis based on the core-genome alignment demonstrated the evolutionary relationships among all isolates and selected international reference genomes. The phylogeny resolved two major clades. **Clade I** comprised four reference genomes (154NSRL, CT18, WGS1146, and R19) together with one clinical isolate (ST40), indicating close phylogenetic relatedness between this isolate and globally circulating reference strains. **Clade II** contained the remaining clinical isolates recovered in the present study, forming a distinct cluster separate from the international reference genomes. Within this clade, several well-supported sub-lineages were observed. Isolates ST36 and ST72 formed a closely related cluster, while ST41, ST60, and ST62 grouped together within another highly supported lineage. Similarly, ST41, ST42, ST60, and ST62 exhibited close phylogenetic relationships, reflecting the high genomic similarity among these isolates. Pairwise core-genome SNP distances confirmed these relationships: isolates within Clade II differed by 0–9 SNPs, with ST-41/ST-60/ST-62, ST-59/ST-73/ST-84, and ST-36/ST-72/ST-75 being identical across the core genome (0 SNPs), whereas ST40 differed from the closest reference genome (R19) by only three SNPs, consistent with its grouping in Clade I (Figure 6B). The phylogenetic topology was consistently observed in tree visualization (Figure 6A). Re-analysis of the recombination-masked alignment supported the separation of clinical isolates from CT18 and 154NSRL, but the placement of the two remaining reference genomes (R19, WGS1146) relative to the clinical cluster was not consistent between the unmasked and recombination-corrected trees, reflecting limited phylogenetic signal at these deeper nodes (23 parsimony-informative sites remained after masking). This indicates that some of the apparent clustering of international reference genomes in Clade I should be interpreted with appropriate caution, as it may be partly influenced by ancestral recombination rather than shared recent descent.

## 4. Discussion

In the present study, we integrated phenotypic antimicrobial susceptibility testing (AST) with whole-genome sequencing (WGS) to characterize the antimicrobial resistance, virulence determinants, genomic composition, and phylogenomic relationships of *S. Typhi* isolates recovered in Peshawar. The genomic findings indicate that the XDR *S. Typhi* isolates in this collection belong predominantly to the ST1 lineage and share a highly conserved antimicrobial resistance and virulence profile. However, the limited number of sequenced isolates restricts broader inference regarding the genomic diversity of XDR *S. Typhi* in Pakistan.

The high prevalence of XDR *S. Typhi* isolates observed in the present study (60.3%) is consistent with the substantial burden of antimicrobial-resistant typhoid reported in Pakistan. Khan et al. (2025) identified XDR typhoid as an important antimicrobial resistance concern in the country [30], while Sohail et al. (2024) [37] reported the dissemination of ceftriaxone-resistant *S. Typhi* carrying the ESBL gene *bla*CTX-M-15 in different regions of Pakistan. The observed resistance to ciprofloxacin, ampicillin, chloramphenicol, ceftriaxone, and co-trimoxazole was consistent with the XDR phenotype and with previously reported resistance patterns in Pakistan [37]. In the present study, *S. Typhi* isolates were more frequently obtained from males (59.5%) and children aged 6–15 years (50.8%). This age distribution is comparable to findings from Northern Punjab, where Khalid et al. (2026) reported that approximately 60% of XDR typhoid cases occurred among children aged 5–10 years [38]. Fatima et al. (2023) similarly reported a higher frequency of typhoid infection among school-aged children in a genomic surveillance study from Balochistan [23]. However, these demographic patterns should be interpreted cautiously because the present study was not designed to determine factors underlying age- or sex-specific differences in *S. Typhi* infection.

Phenotypic AST demonstrated high resistance among the 252 *S. Typhi* isolates, with resistance rates of 98.8% for ciprofloxacin, 92.1% for ampicillin, 91.7% for chloramphenicol, and 88.9% for ceftriaxone. This pattern was consistent with the XDR phenotype and with previous reports from Pakistan describing high resistance to these antimicrobial classes. Sohail et al. (2024) reported widespread ceftriaxone-resistant *S. Typhi* carrying the ESBL gene *bla*CTX-M-15 among clinical isolates from different regions of Pakistan [37]. In contrast, azithromycin and meropenem retained high in vitro activity, with susceptibility rates of 94.0% and 100%, respectively, consistent with previous reports from Pakistan [23,39]. These findings highlight the continued importance of susceptibility monitoring for the remaining active agents used against XDR typhoid.

WGS of the 14 XDR *S. Typhi* isolates provided further information on their genomic characteristics and resistance determinants. All sequenced isolates belonged to ST1, indicating a conserved sequence-type background within the study collection. This finding is consistent with previous genomic studies identifying ST1 among XDR *S. Typhi* isolates circulating in Pakistan. No plasmid replicons were detected in the sequenced genomes. However, the absence of detectable plasmid replicons does not establish chromosomal localization of the resistance genes, as short-read assemblies may not resolve mobile genetic elements or their genomic context. Long-read sequencing and analysis of resistance-gene flanking regions would be required to resolve their precise genomic locations [30]. Compared with previous genomic studies from Pakistan, the present study combines phenotypic AST with genome-wide analyses of antimicrobial resistance, virulence-associated genes, pan-genome structure, and phylogeny in XDR *S. Typhi* from Peshawar. The conserved ST1 background, resistome, and virulome, together with limited gene-content variation, indicate substantial genomic conservation among the 14 sequenced isolates; however, their limited number and regional sampling restrict broader conclusions regarding the national population structure. Resistome analysis showed a highly conserved profile among the 14 XDR *S. Typhi* isolates, with *bla*CTX-M-15, *bla*TEM-1, *catA1*, *dfrA7*, *qnrS1*, and *aac(6′)-Iy* detected across all genomes. The universal presence of *bla*CTX-M-15 is consistent with previous reports from Pakistan, where this ESBL gene has been frequently detected among XDR *S. Typhi* isolates [30,37]. The detection of *qnrS1* and fluoroquinolone target-gene mutations was also consistent with previous genomic reports [30]. Together, these findings demonstrate a conserved resistance profile among the sequenced isolates. As noted above, the absence of plasmid replicons does not by itself establish chromosomal localization of these resistance genes.

Despite the extensive antimicrobial resistance observed, major virulence-associated genes were conserved across all 14 XDR *S. Typhi* isolates. Genes associated with SPI-1, SPI-2, the Vi capsule-associated *vexABCDE* locus, and typhoid toxin-associated determinants were detected across all sequenced isolates. This pattern is consistent with previous genomic studies from Pakistan [23,30]. However, gene detection alone does not establish gene expression, virulence, or fitness. Therefore, the conserved virulence-associated gene profile should be interpreted as evidence of genetic conservation rather than functional equivalence among isolates. Pan- and core-genome analyses showed limited variation in gene content among the 14 sequenced XDR *S. Typhi* isolates. Phylogenomic analysis further demonstrated close relationships among most clinical isolates, while ST40 clustered with selected international reference genomes. Together, these findings indicate substantial genomic conservation within the sequenced collection. However, the limited sample size, single-region sampling, and use of short-read sequencing restrict broader inference regarding population diversity and the genomic context of resistance determinants. In addition, the absence of phenotypic polymyxin susceptibility testing prevents direct interpretation of the detected *pmrA*/*pmrB* variants in relation to polymyxin resistance.

## 5. Conclusions

This study characterized the phenotypic and genomic features of XDR *S. Typhi* isolates from Peshawar, northwestern Pakistan. All 14 sequenced isolates belonged to ST1 and showed a highly conserved resistome and virulence-associated gene profile, with limited pan-genome expansion and genomic variation within the study collection. These findings provide genomic evidence of substantial conservation among the sequenced XDR isolates but do not, by themselves, establish ongoing transmission or clonal expansion. Continued genomic surveillance, combined with antimicrobial stewardship, vaccination, and effective infection-control measures, is warranted to monitor XDR *S. Typhi* in Pakistan.

### Limitations of the Study

This study has two main limitations. First, *S. Typhi*-specific molecular confirmation or serological agglutination was not performed; identification was based on phenotypic, biochemical, and 16S rRNA methods, which have limited discriminatory power among closely related *Salmonella* serovars. Second, WGS was performed on only 14 of the 152 XDR isolates, selected based on non-duplication and DNA availability rather than a formal epidemiological sampling strategy. Therefore, the genomic, resistome, virulome, pan-genome, and phylogenomic findings may not fully represent the diversity of XDR *S. Typhi* in the wider study population or other regions of Pakistan. Larger, geographically diverse studies using *S. Typhi*-specific confirmation are needed to better assess genomic diversity and epidemiological relationships.

## Figures and Tables

**Table 1:** Demographic characteristics of patients and phenotypic classification of *S. Typhi* isolates (n = 252).

Variable	Category	n (%)	*p*-Value
Gender	Male	150 (59.5)	0.001
	Female	102 (40.5)	
Age group (years)	≤5	52 (20.6)	0.013
	6–15	128 (50.8)	
	≥16	72 (28.6)	
Phenotypic classification	Susceptible	27 (10.7)	<0.001
	MDR	73 (29.0)	
	XDR	152 (60.3)	

Note: *p*-values represent two-sided Pearson’s chi-square goodness-of-fit tests comparing the observed frequencies within each categorical variable with equal expected proportions. The null hypothesis was that the categories were equally distributed. These tests were not intended to assess associations between demographic characteristics and phenotypic resistance classification. A two-sided *p* < 0.05 was considered statistically significant.

**Table 2:** Distribution of antimicrobial resistance determinants identified among whole-genome sequenced XDR *Salmonella enterica* serovar *Typhi* isolates (n = 14).

Category	Antimicrobial Class	Resistance Determinant(s)	Frequency, n (%)
Acquired AMR genes	β-lactams	*blaCTX-M-15*, *blaTEM-1*	14 (100)
	Aminoglycosides	*aac(6′)-Iy*	14 (100)
	Aminoglycosides	*aph(6)-Id*	3 (21.4)
	Chloramphenicol	*catA1*	14 (100)
	Trimethoprim	*dfrA7*	14 (100)
	Sulfonamides	*sul1*	2 (14.3)
Chromosomal resistance-associated determinants	Fluoroquinolone-associated	*gyrA*, *gyrB*, *parC*, *parE*	14 (100)
	Antifolate-associated	*folP*	14 (100)
	Polymyxin-associated	*pmrA*, *pmrB*	14 (100)
Regulatory/efflux-associated determinants	Multidrug efflux/regulation	*soxS*, *acrA*, *emrB*, *emrR*, *mdfA*, AcrAB-TolC	14 (100)

**Table 3:** Non-synonymous mutations identified in antimicrobial resistance-associated genes of whole-genome sequenced XDR *Salmonella enterica* serovar *Typhi* isolates.

Gene	Protein Substitution	Resistance-Associated Functional Category
*soxS*	p.D19N	Global regulator/multidrug efflux
*gyrA*	p.A119T	Fluoroquinolone resistance
*parC*	p.Phe173Ile	
*pmrA*	p.G211R, p.T89S	Polymyxin resistance
*pmrB*	p.G73S, p.I83V, p.M15T	

**Table 4:** Distribution of major virulence-associated gene clusters identified among whole-genome sequenced XDR *Salmonella enterica* serovar *Typhi* isolates (n = 14).

Virulence Category	Representative Genes	Frequency, n (%)
Fimbrial adhesion	*csgA–G*, *bcfA–D*, *fimC*, *fimD*, *fimF*	14 (100)
SPI-1 type III secretion system	*invA–I*, *orgA–C*, *prgH*, *prgK*, *sicA*, *spaO–S*	14 (100)
SPI-2 type III secretion system	*ssaC–V*, *sscA–B*, *sseB*, *sseE*, *ssrA*	14 (100)
Effector proteins	*sipA–C*, *pipB*, *sifB*	14 (100)
Vi capsular polysaccharide	*vexABCDE*-Vi capsule export/transport	14 (100)
Non-fimbrial adhesion	*misL*	14 (100)
Macrophage survival	*mig-14*	14 (100)
Magnesium and iron acquisition	*mgtB*, *mgtC*, *iroN*	14 (100)
Typhoid toxin	*cdtB*-typhoid toxin component	14 (100)

## Data Availability

The datasets generated during the current study have been deposited in the National Center for Biotechnology Information (NCBI) Sequence Read Archive (SRA) under BioProject accession number PRJNA1355065. The data are publicly accessible at https://www.ncbi.nlm.nih.gov/bioproject/PRJNA1355065/ (accessed on 5 November 2025).

## Supplementary Materials

The following supporting information can be downloaded at: https://www.mdpi.com/article/10.3390/microorganisms14102225/s1, Table S1: Branch lengths and ultrafast bootstrap (UFBoot) support values for the core-genome maximum-likelihood phylogeny (Figure 6); Table S2: Polymerase chain reaction parameters and primer sequence for identifying *S. Typhi* isolates; Table S3: Isolate-level phenotypic resistance profile and antimicrobial resistance determinants among the 14 whole-genome sequenced XDR Salmonella enterica serovar Typhi isolates. Table S4: Detection of virulence genes in the study samples (n = 14). Table S5: Quality matrices/parameters distribution of *S. Typhi* isolates. Table S6: Phenotypic antimicrobial susceptibility profile of non-duplicate XDR Salmonella enterica serovar Typhi isolates selected for whole-genome sequencing (n = 14). Table S7: Pairwise core-genome SNP distances (primary/full core-genome alignment, matches Figure 6).
